# Analysis of bowel function, urogenital function, and long-term follow-up outcomes associated with robotic and laparoscopic sphincter-preserving surgical approaches to total mesorectal excision in low rectal cancer: a retrospective cohort study

**DOI:** 10.1186/s12957-022-02631-0

**Published:** 2022-05-27

**Authors:** Bo Yang, Shangxin Zhang, Xiaodong Yang, Yigao Wang, Deguan Li, Jian Zhao, Yongxiang Li

**Affiliations:** Department of General Surgery, the First Affiliated Hospital of Anhui Medical University, 218 Jixi Road, Hefei, China

**Keywords:** Robotic surgery, Sphincter-preserving surgery, Total mesorectal excision, Low rectal cancer

## Abstract

**Objective:**

The present study comparatively analyzed short-term clinical effectiveness and long-term follow-up endpoints associated with robotic-assisted sphincter-preserving surgery (RAS) and laparoscopic-assisted sphincter-preserving surgery (LAS) when used to treat low rectal cancer.

**Method:**

Within such a single-center retrospective cohort analysis, low rectal cancer patients that underwent RAS (*n*=200) or LAS (*n*=486) between January 2015 and beginning of July 2018 were enrolled.

**Results:**

The mean operative durations in the RAS and LAS cohorts were 249±64 min and 203±47 min, respectively (*P*<0.001). Temporary ileostomy rates in the RAS and LAS cohorts were 64.5% and 51.6% (*P* = 0.002). In addition, major variations across such cohorts regarding catheter removal timing, time to liquid intake, time to first leaving bed, and length of hospitalization (all *P*<0.001). This distal resection margin distance within the RAS cohort was diminished in comparison to LAS cohort (*P*=0.004). For patients within the LAS cohort, the time required to recover from reduced urinary/female sexual function was > 6 months post-surgery (*P*<0.0001), whereas within the RAS cohort this interval was 3 months (*P*<0.0001). At 6 months post-surgery, male sexual function within RAS cohort was improved in comparison to LAS cohort (*P*<0.001). At 6 months post-surgery, Wexner scores revealed similar results (*P*<0.001). No major variations within overall or disease-free survival were identified across these cohorts at 3 or 5 years post-surgery.

**Conclusion:**

Robotic sphincter-preserving surgery is a safe and effective surgical technique in low rectal patients in terms of postoperative oncological safety and long-term endpoints. And the RAS strategy provides certain additional benefits with respect to short-term urogenital/anorectal functional recovery in treated patients compared to LAS.

**Supplementary Information:**

The online version contains supplementary material available at 10.1186/s12957-022-02631-0.

## Introduction

Rectal cancer is a highly prevalent tumor type, affecting the gastrointestinal system, with high incidence rates in many populations throughout the globe in epidemiological studies [1–3]. Drastic resection typically represents the best therapeutic approach in rectal cancer patients. Due to initial proposal of minimally invasive surgery as a therapeutic approach [4, 5], laparoscopic surgery (LAS) has emerged as an increasingly popular approach to rectal cancer treatment [6], with several randomized controlled trials (RCTs) and comparative studies having underscored the safety and effectiveness of LAS-based rectal resection procedures, which induce only mild trauma and are associated with fewer short-term complications and more rapid patient recovery as compared to open-surgery [7–12]. Within patients diagnosed with low rectal cancer, abdominoperineal resection (APR) is a conventional treatment approach [13], but owing to advances in surgical techniques such as the development of the total mesorectal excision (TME) procedure [14], surgeons have been increasingly attentive to preserving the sphincter when possible. As the quality of life following APR tends to be lower, many patients elect to undergo sphincter-preserving treatment where possible [15, 16]. However, conducting LAS-based sphincter-preserving surgical procedures in low rectal cancer patients with a narrow pelvic cavity can be challenging owing to technical issues including the rigidity of surgical instruments, the restricted 2D-based motion range and visual field, camera platform instability, and tremors on the part of surgical assistants [17]. Therefore, due to these technical issues, the laparoscopic approach may increase rates of conversion to open-surgery for low rectal cancer patients. Laparascopic TME and conversion to open-surgery were related to increased urogenital dysfunction incidence relative to that for patients undergoing conventional open TME resection within the MRC CLASICC trial [18–20].

Robot-assisted surgery (RAS) has emerged as a novel approach for overcoming most technical limitations associated with LAS procedures by improving instrument flexibility, eliminating any tremor on behalf of the operator, offering advanced stereoscopic vision, and providing more comfortable and ergonomic operating conditions [21, 22], all of which may be conducive to the sphincter and pelvic autonomic nerve conservation. In the ROLARR RCT, no significant differences in rates of conversion to open laparotomy were observed when comparing patients that underwent LAS and RAS [23]. Moreover, this trial examined short-term effectiveness and safety endpoints associated with RAS when used to conduct high (upper rectal) resection, low (total rectal) resection, anterior resection, and abdominoperineal (rectum and perineum) resection. Notwithstanding, a scarcity exists regarding investigations to date that have conducted detailed comparisons of RAS and LAS with respect to short-term postoperative complications and clinical endpoints in low rectal cancer patients [24–26], with many studies that have made such comparisons exhibiting relatively small cohort sizes. As such, drawing conclusions regarding the safety profile and utility for RAS when used to treat low-rectal cancer clinical cases remains challenging.

The present study performed a comparative analysis of short-term and long-term LAS- and RAS-based sphincter-preserving surgical treatment endpoints following radical resection in low-rectal cancer patients, with a particular focus on urogenital and bowel function.

## Methodology

### Investigation design

For the present single-center retrospective cohort investigation, low-rectal cancer cases undergoing LAS (*n*=486) or RAS (*n*=200) from January 1, 2015, to July 1, 2018, were enrolled in this study. The same surgical team conducted all procedures, with all members of this team having appropriate clinical experience and having completed the learning curves for RAS and LAS procedures [27]. The same surgeon additionally performed TME. Surgeons recorded preoperative and postoperative parameters for all patients. In addition, experienced research nurses or doctors interviewed patients with their consent to collect the results of questionnaires pertaining to postoperative urogenital and bowel function. The First Affiliated Hospital of Anhui Medical University ethics committee accepted this investigation, with all patient participants providing informed consent (Reference number: Quick-PJ 2021-15-34).

### Study population and treatments

Patients eligible for inclusion were those meeting the following criteria: (1) individuals ≥ 18 years of age histologically diagnosed with rectal adenocarcinoma; (2) patients with tumors < 6 cm from anal verge as detected via rectal magnetic resonance imaging (MRI) or sigmoidoscopy; (3) patients with clinical T stage (cT) < T4a, negative circumferential margins (CRMs) and without distant metastases (M1) or invasion of neighboring organs/tissue (T4a or T4b) as detected via rectal MRI or abdominal pelvic enhanced computed tomography (CT) scans; and (4) patients with an ASA classification<IV and without surgical contra-indications. Surgical approaches were made based on joint decisions made through discussions between surgeons and patients

Routine preoperative procedures used to evaluate all patients included digital rectal examination (DRE), sigmoidoscopic biopsy, rectal/hepatic MRI scans, or abdominal pelvic enhanced CT scans. In addition, serum albumin, hemoglobin, and tumor marker levels were analyzed. Those patients diagnosed with locally advanced disease (cT4aN1-2M0) via appropriate imaging scans were administered neoadjuvant chemoradiotherapy to decrease tumor burden and to improve the odds of a good postoperative prognosis. Neoadjuvant chemoradiotherapy consisted of 2–3 3-week cycles of oxaliplatin or raltitrexed in combination with capecitabine and radiotherapy (50.4 Gy). At 6–8 weeks after neoadjuvant chemoradiotherapy, patients underwent these same preoperative examinations. Those patients meeting inclusion criteria for drastic resection then were subjected to RAS or LAS treatment.

All RAS interventions were conducted employing the Da Vinci Si surgical system (Intuitive Surgical). Total mesorectal excision (TME) and pelvic autonomic nerve preservation were conducted for all clinical cases, with standard high-quality TME techniques being used for all LAS and RAS procedures. Whether anastomosis was conducted via stapling or hand-sewing was determined based upon tumor location and intra-operative conditions. The tension of the anastomosis and splenic flexus mobilization was assessed, with temporary ileostomy being performed at the discretion of the operating surgeon. Ileostomy closure was conducted at 3–6 months postoperatively or following the completion of post-surgical adjuvant chemotherapy or chemo-radiotherapy.

### Data and materials

Investigation datasets pertaining to enrolled participants were obtained from an electronic patient record database. Analyzed patient profiles included age, sex, body mass index (BMI), pre-operative comorbid diabetes, ASA classification, serum hemoglobin, serum CEA, serum albumin, serum CA199, tumor proximity to the anal verge, clinical TNM stage, and neoadjuvant chemoradiotherapy treatment. Analyzed intra-operative endpoints consisted of surgery duration, hemorrhage level, blood transfusion requirements, and whether patients underwent conversion to laparotomy and/or temporary ileostomy. Analyzed post-surgical recovery endpoints included timeframe for initial mobility sigms (first leaving bed), timings for initial flatus/liquid diet/catheter removal, total drainage volume, the duration of hospitalization, and visual analog scale (VAS) scores on days 1–3. Post-surgery issues assessed in less than 30 days after surgery were stratified as grades I–V as per the Clavien-Dindo classification system [28]. Analyzed pathology dataset outcomes mainly consisted of tumor size, histological type, lymph node status, nerve invasion, vascular invasion, distal resection margin (DRM), positive circumferential resection margin (CRM; > 1 mm [29]), and pathologic T/N stage. The total costs included operative costs and hospitalization costs. Robotic costs also included the costs of the maintenance of the robotic device.

### Postoperative follow-up analyses of patient urogenital and bowel function

A standard questionnaire developed based upon the International Prostatic Symptom Score (IPSS) was used to assess postoperative urogenital function in enrolled patients. This questionnaire consisted of 7 questions pertaining to voiding ability, with scores for individual items being summed. Total scores of 0–7, 8–19, and 20–35 corresponded to mild, moderate, and severe symptoms, accordingly.

Male sexual function was assessed founded upon the international index of erectile function (IIEF) questionnaire, consisting of 15 items assessing erectile function, libido, orgasm, overall sexual gratification, and intercourse gratification. Female sexual function was analyzed using the Female Sexual Function Index (FSFI), which assessed items pertaining to pain, lubrication, arousal, desire, orgasm, and satisfaction. The items for each of these scales were summed to determine the total IIEF or FSFI score for each patient.

Postoperative bowel function was assessed using Wexner scores consisting of 5 items pertaining to gaseous/liquid/solid incontinence, pad-wearing, together with lifestyle alterations. Individual scores were summed together, with scores of 0, 1–8, 9–14, and 15–20, respectively, corresponding to normal functionality, and mild, moderate, or complete incontinence. Other evaluated follow-up endpoints included the use of adjuvant postoperative therapies, localized recurrence, distant metastases, and 1, 3, 5–year overall survival (OS) and disease-free survival (DFS).

### Statistical analysis

SPSS 22.0 (IL, US) was used for all statistical analyses. Categorical variables were compared through chi-squared tests or Fisher’s exact test. Quantitative variables were compared through Student’s *t* tests or Mann-Whitney *U* tests and reflected mean±SD or the median with the range when parametric or non-parametric, respectively. Patient OS and DFS at 1, 3, and 5 years post-surgery were assessed via the Kaplan-Meier method. Potential predictors of patient OS and DFS were identified via Cox regression analyses. *P* < 0.05 was the threshold of significance.

## Results

From January 2015 to July 2018, 686 total low-rectal cancer patients meeting the criteria for treatment in our gastrointestinal surgery department were enrolled in this study, of whom 200 and 486 underwent LAS- and RAS-based sphincter-preserving surgical procedures, respectively. The preoperative clinical profiles for such cases are compiled within Table 1. A significantly lower distance from the anal verge was observed within the RAS cohort as compared to the LAS cohort (5.06±0.84 cm vs 5.66±0.53 cm, *P*<0.001). There were no major variations across these two cohorts regarding age, sex, ASA classification, diabetes incidence, serum CEA levels, serum CA199 levels, serum hemoglobin, plasma albumin, or NRS 2002 scores. In addition, clinical T stage, N stage, and TNM staging distributions were comparable between these cohorts, as were the proportions undergoing preoperative neoadjuvant chemoradiotherapeutic treatment. The total operative cost was elevated within the RAS cohort (53922±14290 ¥ vs 48522±17466 ¥, *P*<0.001).

**Table 1:** Clinical characteristics

Variables	RAS	LAS	P
Total clinical cases, *n*	200	486	
Gender (male, %)	121 (60.5)	302 (62.1)	0.688
Average age (mean, SD)	58.4±11.8	59.8±11.5	0.149
Body mass index (mean, SD)	23.1±3.1	22.9±3.5	0.402
ASA classification			
I(%)	26 (13)	52 (10.7)	0.335
II(%)	134 (67)	353 (72.6)	
III(%)	40 (20)	81 (16.7)	
NRS2002 score (median, IQR)	2 (2,3)	2 (2,3)	0.408
Diabetes (yes, %)	26 (13)	54 (11.1)	0.484
Level of hemoglobin(g/L, mean, SD)	130.9±15.4	129.5±15.3	0.263
Level of plasma albumin(g/L, mean, SD)	43.2±7.1	42.7±3.6	0.163
Level of CEA (≥5 ng/ml, %)	49 (24.5)	154 (31.7)	0.061
Level of CA199 (≥36 u/ml, %)	18 (9)	58 (11.9)	0.266
Distance from anal edge (cm, mean, SD)	5.06±0.84	5.66±0.53	<0.0001
MRI cT stage, *N* (%)			
T1 (%)	26 (13)	59 (12.1)	0.738
T2 (%)	117 (58.5)	274 (56.4)	
T3 (%)	57 (28.5)	153 (31.5)	
MRI cN stage, *N* (%)			
N0 (%)	126 (63)	336 (69.1)	0.297
N1 (%)	43 (21.5)	87 (17.9)	
N2 (%)	31 (15.5)	63 (13)	
MRI cTNM stage, *N* (%)			0.464
I(%)	99 (49.5)	250 (51.4)	
II(%)	39 (19.5)	107 (22)	
III(%)	62 (31)	129 (26.5)	
Neoadjuvant chemoradiotherapy (%)	21 (10.5)	43 (8.8)	0.441
Total hospitalization costs (¥), mean (SD)	53,922±14290	48,522±17466	<0.0001

*RAS* robotic-assisted surgery cohort, *LAS* laparoscopic-assisted surgery cohort, *NRS* nutritional risk screening, *SD* standard deviation, *IQR* interquartile range, *MRI* magnetic resonance imaging

### Short-term endpoints

Intra-surgical details and postoperative recuperation parameters for enrolled patients are compiled in Table 2. Conversion to open surgery was performed for just two cases (0.4%) within the LAS cohort, and one case due to intraoperative bleeding and the other being due to the patient exhibiting a narrow pelvis and pelvic adhesions. The operative duration within the RAS cohort was markedly longer in comparison to the LAS cohort (249±64 min vs. 203±47 min, *P*<0.001), while projected intra-surgical hemorrhage with markedly greater within LAS cohort (95±33 ml vs. 82±49 ml, *P*=0.001). Blood transfusions were respectively required for 8 patients (4%) and 18 patients (3.7%) within RAS and LAS cohorts, with no variations across cohorts (*P*>0.05). Overall, 129 (64.5%) and 251 (51.6%) patients underwent temporary ileostomy within RAS and LAS cohorts, accordingly (*P*<0.05). No clinical cases from either cohort experienced intraoperative adverse events. Timeframe for initial flatus and liquid diet were diminished within the RAS cohort [2 (1,3) vs 3 (2,3), *P*<0.0001; 3 (2,4) vs 4 (3,4), *P*<0.0001, accordingly]. Similarly, patients in the RAS cohort exhibited a shorter time to first leaving bed as compared to patients in the LAS cohort [2 (2,3) vs 3 (2,3), *P*<0.001]. Timeframe for catheter removal within the RAS cohort was also significantly shorter [4.9±1.2 vs 5.2±1.3, *P*=0.004], as was the average duration of hospitalization (9.5±4.6 days vs 11.3±5.9 days, *P*<0.001). VAS scores, drainage cube duration, and total drainage volume did not differ significantly between cohorts.

**Table 2:** Intraoperative, postoperative recovery, and pathological endpoints

Variables	RAS (*n*=200)	LAS (*n*=486)	*P*
Operative time (min, mean, SD)	249±64	203±47	<0.0001
Intraoperative blood loss, mean (SD), ml	82±49	95±32	0.001
Blood transfusion (yes, %)	8 (4)	18 (3.7)	0.765
Conversion to laparotomy	0 (0)	2 (0.4)	1
Temporary ileostomy (yes,%)	129 (64.5)	251 (51.6)	0.002
The leaving bed time (days, median, IQR)	2 (2,3)	3 (2,3)	<0.0001
Time to first flatus, days, median (P25, P75, IQR)	2 (1,3)	3 (2,3)	<0.0001
Time to liquid diet, days, median (P25, P75, IQR)	3 (2,4)	4 (3,4)	<0.0001
VAS score, median (P25, P75, IQR)			
Day 1	2 (1,2)	2 (1,2)	0.624
Day 2	1 (1,2)	1 (1,2)	0.54
Day 3	1 (1,1)	1 (1,1)	0.738
Removal time of catheter, days, mean (SD)	4.9±1.2	5.2±1.3	0.004
Volume of drainage, ml, mean (SD)	209±63	222±100	0.083
The drainage of cube duration, days, mean (SD)	5.7±1.3	5.9±1.6	0.168
Postoperative length of stay, days, mean (SD)	9.5±4.6	11.3±5.9	<0.0001
Overall complications, *n* (%)	32 (16)	86 (17.7)	0.593
Anastomotic leakage (%)	8 (4)	24 (4.9)	0.596
Anastomotic and abdominal bleeding (%)	2 (1)	5 (1)	1
Wound infection (%)	2 (1)	4 (0.8)	1
Abdominal infection (%)	1 (0.5)	5 (1)	0.677
Pulmonary infection (%)	3 (1.5)	10 (2.1)	0.765
Urinary infection (%)	1 (0.5)	5 (1)	0.677
Urinary retention (%)	7 (3.5)	20 (4.1)	0.706
Cardiovascular accident (%)	1 (0.5)	1 (0.2)	0.498
Venous thromboembolism (%)	0 (0)	1 (0.2)	1
Ileus (%)	7 (3.5)	11 (2.3)	0.357
Clavien-Dindo classification			
I(%)	10 (5)	24 (4.9)	0.887
II(%)	16 (8)	52 (10.7)	
III(%)	4 (2)	6 (1.2)	
IV(%)	1 (0.5)	2 (0.4)	
V(%)	1 (0.5)	2 (0.4)	
Tumor size, cm, mean (SD)	3.95±1.30	4.05±1.33	0.345
Tumor differentiation, *n* (%)			
Well differentiated adenocarcinoma	10 (5)	27 (5.6)	0.319
Moderately differentiated adenocarcinoma	160 (80)	361 (74.3)	
Poorly differentiated adenocarcinoma	18 (9)	49 (10.1)	
Mucinous adenocarcinoma	12 (6)	49 (10.1)	
Number of harvested lymph nodes, mean (SD)	13.6±3.7	14.0±3.7	0.169
Vascular invasion, *n* (%)	118 (59)	291 (59.9)	0.832
Nerve invasion, *n* (%)	63 (31.5)	126 (25.9)	0.138
Tumor deposit, *N* (%)	55 (27.5)	137 (28.2)	0.855
Distal resection margin (cm, median, IQR)	1.4 (1.1,1.875)	1.6 (1,2)	0.004
Positive circumferential resection margin, *n* (%)	1 (0.5)	3 (0.6)	1
Quality of the mesorectal excision R0 resection, *n* (%)	200 (100)	484 (99.6)	1
pT stage, *N* (%)			
Tis	6 (3)	12 (2.5)	0.569
T1	24 (12)	49 (10.1)	
T2	58 (29)	120 (24.7)	
T3	106 (53)	292 (60.1)	
T4a	6 (3)	13 (2.7)	
T4b			
pN stage, *N* (%)			
N0	121 (60.5)	277 (57)	0.48
N1a	21 (10.5)	46 (9.5)	
N1b	24 (12)	59 (12.1)	
N1c	13 (6.5)	25 (5.1)	
N2a	11 (5.5)	35 (7.2)	
N2b	10 (5)	44 (9.1)	
pTNM stage, *N* (%)			
I	67 (33.5)	132 (27.2)	0.251
II	56 (28)	150 (30.9)	
III	77 (38.5)	204 (42)	

*VAS* visual analog scale

Postoperative complications for the patients in these two treatment cohorts were next evaluated (Table 2). In total, 118 complications were reported, including 32 (16%) and 86 (17.7%) within RAS and LAS cohorts, accordingly (*P*= 0.593). These adverse events included 1 case of death due to a cardiovascular accident within the RAS cohort (0.5%), and 2 deaths within the LAS cohort (0.4%), one of which was the result of cardiovascular accident and the other was associated with anastomotic leakage and consequent multiple organ failure (*P*>0.05). Post-surgical urinary retention within LAS cohort was increased relative to that in the RAS cohort (4.1 vs 3.5%, *P*=0.706), though such a variation failed to achieve significance. In addition, complication types demonstrated no variations across both cohorts. Complication severity was classified using the Clavien-Dindo classification system, revealing a lack of major variation across cohorts regarding complication severity (P>0.05). Grade I–II adverse event complications were 81.25% and 88.7% for all complications observed within RAS/LAS cohorts, respectively. Anastomotic leakage occurred in 32 patients (4.7%) in the overall study cohort and was successfully treated via a conservative approach in all but 4 clinical cases within LAS cohort, together with 2 cases within RAS cohort (*P*>0.05). Two pat postoperative ileus necessitating a second operation was reported for 2 and 1 clinical case/s within LAS and RAS cohorts, accordingly, while 1 clinical case within RAS cohort exhibited an intra-abdominal abscess necessitating relaparotomy within 30 days following the initial surgical procedure.

Pathological results for patients included in the present study are compiled in Table 2. The median distal resection margins within RAS/LAS cohorts were 1.4 cm (1.1, 1.9) and 1.6 cm (1, 2), accordingly (*P*<0.05). CRM involvement was observed for 2 clinical cases within LAS cohort (*P*>0.05), with R0 resection being obtained within the remaining patients. No major variations existed across both cohorts regarding tumor size, tumor deposit, histologic differentiation, TNM staging, numbers of harvested lymph nodes, nerve invasion, or vascular invasion status (all *P*>0.05).

### Variations in overall IPSS and Wexner scores

Reported variations within urinary/bowel functions for included patients were next evaluated (Table 3). In total, 169 (84.5%) and 404 (83.1%) clinical cases within RAS and LAS cohorts, accordingly, underwent these assessments. No pre-operative IPSS score differences were observed between these two cohorts (*P*=0.088). IPSS scores had risen substantially as of 30 days postoperatively (*P*>0.05), but were markedly reduced after 90 days postoperatively within RAS cohort [RAS 5, (5,6) vs LAS 7(6~8), *P*<0.001]. This variation between these two cohorts was still evident at 6 months postoperatively (RAS, 5(4~6) vs LAS, 6(4,7), *P*<0.001). Upon reaching 2 years postoperatively, three (1.7%) and nine (2.2%) clinical cases within RAS and LAS cohorts, accordingly, reported suffering from moderate urinary dysfunction (*P*>0.05).

**Table 3:** Analyses of urinary function, bowel function, and sexual function

Variables		Pre-operation median (IQR)	1-month median (IQR)	3-month median (IQR)	6-month median (IQR)	12-month median (IQR)	24-month median (IQR)
**IPSS Score**	RAS (*n*=169)	4 (3~5)	7 (6~9)	5 (5~8)	5 (4~6)	4 (3~5)	4 (3~5)
	LAS (*n*=404)	4 (3~6)	7 (7~9.75)	7 (6~8)	6 (4~7)	4 (3~6)	4 (3~6)
	P valve	0.088	0.067	**<0.001**	**<0.001**	0.186	0.185
**Wexner Score**	RAS (*n*=169)	0 (0,0)			0 (0,1)	0 (0,1)	0 (0,0)
	LAS (*n*=404)	0 (0,0)			1 (1,3)	1 (0,1)	0 (0,0)
	P valve	0.885			**<0.001**	**<0.001**	0.787
**IIEF Score**	RAS (*n*=46)	59.4±4.5		20.2±4.2	38.5±4.5	46.4±5.9	51.4±6.2
	LAS (*n*=75)	59.1±4.9		19.1±4.1	34.5±5.8	41.0±5.6	50.5±5.8
	P valve	0.707		0.199	**<0.001**	**<0.001**	0.406
**FIFS Score**	RAS (*n*=20)	25.1±3.9		16.6±3.7	19.4±3.9	21.3±4.2	23.0±4.3
	LAS (*n*=55)	24.5±3.1		14.2±2.5	16.1±2.7	19.0±3.1	22.1±3.7
	P valve	0.484		**0.003**	**<0.001**	**<0.001**	0.389

*IPSS* International Prostate Symptom Score, *IIEF* the International Index of Erectile Function, *FIFS* the Female Sexual Function Index

In line with the IPSS scores, Wexner scores initially rose after surgery before gradually declining over the course of recovery. At 6 months postoperatively, the scores within RAS cohort were markedly reduced in comparison to within LAS cohort [RAS 0 (0.1) vs 1(1,3), *P*<0.001]. While scores within RAS cohort had recovered to normal levels as of 12 months post-surgery, such recovery took over 1 year within LAS cohort [RAS 0 (0,1) vs LAS 1 (0,1), *P*<0.001].

### Changes in total IIEF and FIFS scores

IIEF score analyses were conducted for the 121 male clinical cases enrolled in this study, of whom 75 and 46 were enrolled within LAS and RAS cohorts, accordingly (RAS 46, LAS 75) (Table 3). Mean IIEF scorings at 6 and 12 months post-surgery within RAS cohort were significantly elevated relative to those within LAS cohort [RAS 38.5±4.5 vs LAS 34.5±5.8, *P*<0.001; RAS 46.4±5.9 vs 41.0±5.6, *P*<0.001, accordingly]. Erectile dysfunction was reported by 1 and 2 clinical cases within RAS and LAS cohorts, accordingly, at 2 years after surgery.

In total, sexual function analyses were conducted for 75 clinical cases included in this study, including 55 and 20 within LAS and RAS cohorts, accordingly (Table 3). Significantly more rapid recuperation of sexual function was observed within RAS cohort at 90 days post-operation as compared to the LAS cohort (RAS 16.6±3.7 vs LAS 14.2±2.5, *P*=0.003), with a significantly higher mean overall FIFS scorings within RAS cohort as compared to the LAS cohort at 6 and 12 months postoperatively (all *P* <0.05).

### Long-term endpoints

**Fig. 1:**
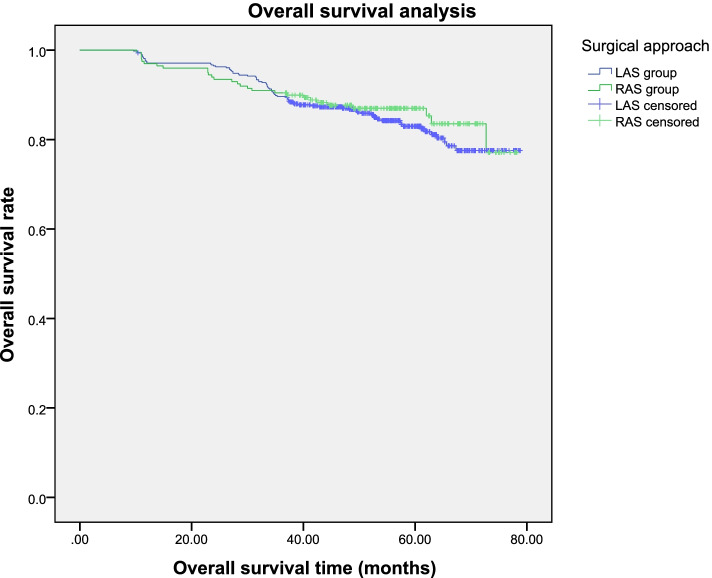
Patients’ 1-, 3-, and 5-year overall survival rates were 97%, 90.5%, and 86.9% within RAS cohort and 97.1%, 89.7% and 85.1% within LAS cohort, accordingly (*P*=0.467)

**Fig. 2:**
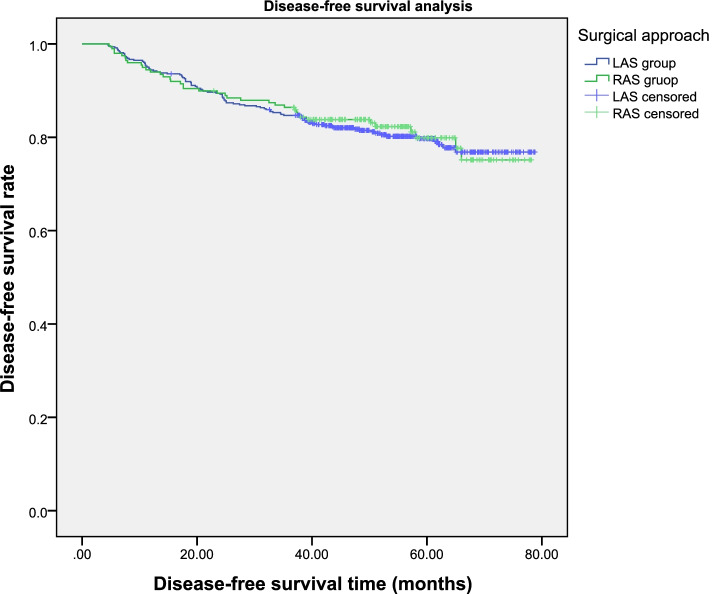
Patients’ 1-, 3-, and 5-year disease-free survival rates were 94.0%, 86.4%, and 80.3% within RAS cohort and 94.4%, 84.7% and 79.2% within LAS cohort, accordingly (*P*=0.746)

The median follow-up duration was 53.2 months and 54.1 months for clinical cases within RAS and LAS cohorts, accordingly. The 1-, 3- and 5-year OS and DFS endpoints within both cohorts were comparable (Figs. 1 and 2 ). In total, distant metastases developed in 69 clinical cases (10.1%), including hepatic (*n*=34), pulmonary (*n*=34), and brain (*n*=1) metastases, having no differences in incidence across both treatment cohorts. Local recurrence proximal to the surgical site was reported for 19 (9.5%) and 50 (10.3%) clinical cases within RAS and LAS cohorts, accordingly (*P*=0.883). Cox regression analyses revealed pT stage, age, positive CRM, and positive lymph nodes (pN+) were significant predictors of postoperative OS (Table 4), with all of these same variables other than age also being significant predictors of patient DFS.

**Table 4:** Cox regression analyses of patient OS and DFS

Univariate and multivariate Cox regression analysis of predictors of OS and DFS								
Variables	Overall survival				Disease-free survival			
	Univariate analysis		Multivariate analysis		Univariate analysis		Multivariate analysis	
	HR (95% CI)	*P*	HR (95% CI)	*P*	HR (95% CI)	*P*	HR (95% CI)	*P*
Surgical approach: robotic surgery vs laparoscopic surgery	1.016 (0.654–1.579)	0.943	NA	NA	0.979 (0.668-1.434)	0.914	NA	NA
Age ≥70 years	1.477 (0.932–2.340)	0.097	**1.592 (1.028**–**2.648)**	**0.037**	0.382 (0.911–2.098)	0.128	NA	NA
Gender: male vs female	1.117 (0.749–1.667)	0.587	NA	NA	1.252 (0.881–1.778)	0.21	1.548 (1.001–2.023)	0.05
Neoadjuvant chemoradiotherapy	1.133 (0.672–1.908)	0.639	NA	NA	1.475 (0.945–2.303)	0.087	NA	NA
Anastomotic leakage	1.717 (0.801–3.680)	0.165	NA	NA	1.557 (0.738–3.283)	0.245	NA	NA
R1 resection	3.594 (0.689–18.759)	0.129	NA	NA	3.197 (0.686–14.899)	0.139	NA	NA
Positive CRM	**8.292 (1.938**–**35.490)**	**0.004**	**14.637 (4.547**–**47.123)**	**<0.0001**	**6.274 (1.641**–**23.994)**	**0.007**	**10.416 (3.261**–**33.266)**	**<0.0001**
T stage: pT1-3 vs pT4a	**2.111 (1.425**–**3.128)**	**<0.0001**	**1.884 (1.327**–**2.674)**	**0.001**	**1.574 (1.131**–**2.191)**	**0.007**	**1.515 (1.136**–**2.019)**	**0.005**
N stage: pN0 vs pN+	**1.521 (1.357**–**1.705)**	**<0.0001**	**1.534 (1.387**–**1.697)**	**<0.0001**	**1.382 (1.251**–**1.527)**	**<0.0001**	**1.411 (1.287**–**1.548)**	**<0.0001**
Postoperative adjuvant chemotherapy	1.877 (0.878–4.015)	0.104	NA	NA	1.203 (0.610–2.372)	0.593	NA	NA
Postoperative adjuvant radiotherapy	0.843 (0.546–1.300)	0.439	NA	NA	0.751 (0.514–1.098)	0.139	NA	NA

*HR* hazards ratio, *CI* confidence interval; an HR<1 is a positive survival predictor; otherwise, it is a negative predictor

## Discussion

Sphincter-preserving surgical approaches to the treatment of low-rectal cancer clinical cases have been widely adopted to date, with recent studies having shown these approaches to be safe and effective [30, 31], although the superiority to APR with respect to patient long-term quality of life is still controversial [32, 33]. Advances in minimally invasive laparoscopic surgical techniques have led to improvements in patient short-term endpoints and reduced postoperative functional recovery durations as compared to those associated with conventional open sphincter-preserving TME procedures in low-rectal cancer clinical cases [34, 35]. Laparoscopic procedures are, however, subject to inherent technical limitations increasing rates for confirmed CRM upon postoperative pathology-based evaluation according to ACOSOG trials [17]. However, a recent large database analysis showed that minimally invasive surgery (MIS) including laparoscopic and robotic approach have superiority in pathologic and clinical outcomes when compared to open approach [36].

Robotic-assisted surgical approaches can prevail over many of the limitations of laparoscopic approaches [21, 22], improving efforts to dissect lymph nodes while preserving vascular integrity and nerve function. Pathological analyses have also demonstrated that a distal margin < 1 cm was not associated with increased risk of tumor development [37], providing a theoretical basis for sphincter-preserving surgical procedures in clinical cases with ultra-low-rectal cancer. Robotic-assisted approaches are thus widely used to conduct sphincter-preserving procedures when treating low-rectal cancer.

Several studies have reported advantages associated with robotic approaches to performing rectal surgery with respect to both intra-surgical endpoints and short-term patient recuperation [23, 38–41], including reductions in blood loss, lower conversion rates, fine-tuned technical performance when performing lateral and inferior mesenteric artery root lymph node dissection, decreases in time to first flatus and liquid intake, together with reduced duration of hospitalization, albeit with higher operative costs and a longer operative duration. The dataset outcomes of this investigation corroborated with outcomes from prior reports.

The two primary concerns when conducting sphincter-preserving TME operations are the postoperative recuperation of bowel and urogenital functions. Heald et al. [14] discussed the critical value for preventing local recurrence while preserving nerve function. Owing to their use of magnified stereoscopic vision, together with an avascular plan when conducting pelvic dissection, robotic surgical techniques can reduce the risk of directly damaging the hypogastric nerve plexus or associated avulsion [42], thereby decreasing the potential for post-surgical urinary and/or sexual dysfunctions. Kim et al. [42] found robotic-TME procedures as associated with the more rapid recuperation of regular voiding function and male sexual function (both sexual desire and erectile function) in comparison to laparoscopic TME. Recent meta-analyses [43–45] have found robotic surgery to similarly offer short-term advantages with respect to postoperative urinary and sexual functional recovery. This study similarly revealed lower postoperative urinary retention rates within 30 days, increased IPSS scorings at 90 days, and enhanced FIFS and IIEF scorings at 90 and 180 days, accordingly, within RAS cohort relative to the LAS cohort, with significant differences between these cohorts. However, no differences were observed with respect to these endpoints at 2 years post-surgery, suggesting that only short-term endpoints differ as a function of operative approach.

Postoperative bowel function recovery is another key concern for clinical cases undergoing surgical treatment for rectal cancer. The impairment of anorectal function is generally the result of internal anal sphincter damage as a result of either direct operative injury or pelvic splanchnic nerve injury [46]. Risk factors known to be associated with post-surgical bowel dysfunctions include peri-surgical radiotherapy together with low anastomosis location [47, 48]. While multiple reports have reported temporary ileostomy to be associated with bowel dysfunction [49, 50], this result is likely attributable to confounding variables including a low anal verge distance and adjuvant radiotherapy. Current evidence suggests that robotic TME can conserve anorectal functionality more effectively in comparison to either open/laparoscopic TME [51]. With this investigation, RAS was associated with the more rapid and successful recovery of bowel function in treated clinical cases within 6 months post-surgery relative to LAS.

Several studies with long-term follow-up timeframes have confirmed safety of robotic surgical approaches to cancer treatment. For example, Feroci et al. [52] assessed the 3-year OS and DFS of middle-low-rectal cancer clinical cases following robotic or laparoscopic surgery and observed no differences, while Cho et al. [53] similarly observed no differences in 5-year OS between these two surgical approaches. Moreover, while Kim et al. [54] detected a trend towards lower 5-year OS rates for laparoscopic surgery as compared to robotic surgery, the differences failed to reach significance. Consistently, within this investigation no major variations existed in 3- or 5-year OS or DFS when comparing the RAS and LAS cohorts. Cox regression analyses additionally indicated that confirmed CRM, pT stage, and pN stage are all negative predictors for OS/DFS within such clinical cases, whereas postoperative adjuvant radiotherapy is associated with better postoperative survival endpoints.

This investigation has a degree of limitations. Firstly, it is not possible to exclude the potential for selection bias or reporting error with respect to these results. Case in point, a considerable degree of clinical cases with ultra-low tumor localization were enrolled within RAS cohort for such analyses relative to the LAS cohort, potentially interfering with analyses of postoperative anorectal function. Second, this was not a randomized trial and is instead the result of a retrospective single-center analysis conducted in a tertiary colorectal treatment center. Intra-surgical endpoints for both sLAS and RAS procedures were inevitably impacted by the experience and skills of the operating surgeon and the associated learning curve for that procedure. There may be substantial differences between these two procedures in this context.

In summary, both robotic and laparoscopic approaches to sphincter-preserving surgery are safe and effective when used to treat low-rectal cancer clinical cases, with the robotic approach offering advantages with respect to both short-term endpoints and the preservation of urogenital and anorectal functions.

## Supplementary Information

**Additional file 1.**

**Additional file 2.**

**Additional file 3.**

## Data Availability

The datasets supporting the conclusion of this article are included within article.
